# Dataset for the analysis of stock price responses to African Swine Fever Outbreaks in China

**DOI:** 10.1016/j.dib.2022.108574

**Published:** 2022-09-05

**Authors:** Tao Xiong, Wendong Zhang, Chen-Ti Chen

**Affiliations:** a Department of Agricultural Economics and Management, Huazhong Agricultural University, China; b Charles H. Dyson School of Applied Economics and Management, Cornell University, United States; c Department of Economics and Center for Agricultural and Rural Development (CARD), Iowa State University, United States

**Keywords:** Abnormal stock returns, African swine fever, Event study, Stock prices

## Abstract

The African Swine Fever (ASF) outbreaks in China since 2018 caused a more than 100 million decline in its hog inventory. Leveraging publicly available announcements of ASF outbreaks and daily stock prices data from 25 major publicly listed hog companies from China and eight major hog exporting countries, we use the event study method to estimate firm-level abnormal stock price responses to China's ASF outbreak announcements for both Chinese and foreign hog companies. This article describes the data used in the research article “A Fortune from misfortune: Evidence from hog firms’ stock price responses to China's African Swine Fever outbreaks” (Xiong et al., 2021). The daily stock price data in this article can be applied to other events that also occurred during the same sample period using a similar event study approach.

**Specifications Table**

**Table:** 

Subject	Economics, Econometrics and Finance
Specific subject area	Statistical methods applied to daily stock price data to estimate the abnormal stock returns for Chinese and foreign hog firms from ASF outbreaks in China.
Type of data	Data Stata Codes Graph Figure
How the data were acquired	All data are downloaded from publicly available sources (see Description of data collection for more).
Data format	Raw Analysed
Description of data collection	ASF announcements in China from August 2018 to September 2019 came from China Ministry of Agriculture and Rural Affairs (http://www.moa.gov.cn/gk/yjgl_1/yqfb/). The ASF announcements detailed the release date, the county-level location and specific site (i.e., pig farm, slaughterhouse, or transport vehicle) of event detection, the number of hogs in inventory, and the number of infected and dead pigs. The release dates were used as event dates in the event study, while the county-level location, the number of hogs in inventory and the number of infected pigs were used as explanatory variables in the Pooled OLS model. Daily market indices and stock price data for China's top 10 publicly listed hog firms and 15 foreign public listed hog firms from eight countries were downloaded from Yahoo Finance (https://finance.yahoo.com).
Data source location	1. China Ministry of Agriculture and Rural Affairs, China (http://www.moa.gov.cn/gk/yjgl_1/yqfb/) 2. Yahoo Finance, United States (https://finance.yahoo.com)
Data accessibility	Repository name: Mendeley Data Data identification number: DOI: 10.17632/tn56tsgpnh.3 Direct URL to data: https://data.mendeley.com/datasets/tn56tsgpnh/3
Related research article	T. Xiong, W. Zhang, C.-T. Chen, A Fortune from misfortune: Evidence from hog firms’ stock price responses to China's African Swine Fever outbreaks, Food Policy. 105 (2021) 120150. https://doi.org/10.1016/j.foodpol.2021.102150

## Value of the Data

• The daily firm-level stock price data contain important information that reflects not only to economic drivers, but often also the non-economic shocks. The stock price data are useful for evaluating firms’ performance in the market in response to both economic and non-economic events. As was the case for African Swine Fever (ASF) outbreaks in China, Xiong, Zhang, and Chen [3] showed that announcements of ASF outbreaks resulted in statistically significant impacts on stock prices for both Chinese and international hog firms, where these hog firms profited from the reduction in Chinese hog inventories due to the epidemic.
• Researchers interested in using stock price as the indicator for firm performance in the market can benefit from these data. The daily stock price data for Chinese and international hog firms allow others to study the effect of any economic and non-economic events that also occurred during the same sample period that might impact stock price movements. For example, researchers interested in the impact of U.S.-China trade war on stock price responses among these companies can use the daily stock price data in this article.
• Both the stock price data for hog firms and the China ASF announcements data presented in this article can not only be reused to explore different aspects of the economic consequences as a result of the ASF outbreaks in China other than for the financial market, but they can also be used for empirical applications of new statistical approaches analysing stock price responses. Researchers that seek to develop new statistical methods to investigate stock price movements can use the presented stock price data in this article for model validation.

## 1 Data Description

Data presented in this article include raw data accessible from the respective public online sources, as well as analysed data that requires necessary statistical program to generate.

The ‘Data’ folder supplied with this article contains three sub-folders (‘Chinese firms and market indices’, ‘Global firms and market indices’, and ‘Cumulative abnormal returns’) and an EXCEL file. The ‘Chinese firms and market indices’ sub-folder contains ten Chinese hog firms’ daily stock prices and four Chinese daily stock market indices. The ‘Global firms and market indices’ sub-folder has fifteen foreign hog firms’ daily stock prices and seven global daily stock market indices. Both the market indices and stock prices were downloaded from Yahoo Finance (https://finance.yahoo.com). The ‘Cumulative abnormal returns’ sub-folder contains the cumulative abnormal returns (CARs), defined as the accumulated difference between the estimated market indices and observed stock returns since each event announcement, for both Chinese and foreign hog firms. The CARs are used for the pooled OLS regression analyses.^1^

The data of ASF event announcements is stored as an EXCEL file under the subfolder ‘Data’. ASF event announcements in China from August 2018 to September 2019 came from China Ministry of Agriculture and Rural Affairs [2] (http://www.moa.gov.cn/gk/yjgl_1/yqfb/). Following each ASF outbreak, MARA announces the release date, the county-level location and specific site (i.e., pig farm, slaughterhouse, or transport vehicle) of event detection, the number of hogs in inventory, and the number of infected and dead pigs. MARA issued a total of 138 ASF announcements during our study period [2]. The release dates were used as event dates in the event study, while the county-level location, the number of hogs in inventory and the number of infected pigs were used as explanatory variables in the Pooled OLS model.

The definition of all variables which appear in the dataset is as follows.

For the variables in the ‘Chinese firms and market indices’ sub-folder:

1. Code: the ticker for that stock
2. Date: the trading date
3. Closing price: the daily closing price for that stock
4. Index: the daily closing index for that market index

For the variables in the ‘Global firms and market indices’ sub-folder:

1. Code: the ticker for that stock or index
2. Date: the trading date
3. Open: the daily opening price for that stock or index
4. High: the daily highest price for that stock or index
5. Low: the daily lowest price for that stock or index
6. Close: the daily closing price for that stock or index
7. AdjClose: the closing price after adjustments for all applicable splits and dividend distributions. Yahoo Finance supplies this variable
8. Volume: the daily trading volume for that stock

For the variables in the ‘Cumulative abnormal returns’ sub-folder:

1. asf_date: the date of the African Swine Fever (ASF) announcements
2. date: the trading dates used in the regression analysis, typically 1-15 days after the ‘asf_date’
3. year: the year of the trading date
4. month: the month of the trading date
5. day: the day of the trading date
6. day_since_announce: the number of days after the ‘asf_date’
7. company: the listed company
8. panel_id: count and mark samples of specific ASF announcements and listed companies
9. event_id: count and mark samples of specific ASF announcements
10. period: We divide the sample period into four groups: (a) August 2018; (b) September 2018 to December 2018; (c) January 2019 to February 2019; and (d) March 2019 to September 2019. The samples in the four groups are marked as 1, 2, 3, and 4, respectively.
11. event: the number of ASF announcements in each group.
12. car: Cumulative abnormal returns calculated from the event study. The formulation of this variable is shown in the ‘Experimental design, materials and methods’ section.
13. trade_volume: the daily trading volume for that stock
14. baidu: the daily number of searches for ‘African Swine Fever’ on Baidu
15. feeder_pig_price: Chinese daily feeder pig prices,
16. hog_inc_share: annual hog breeding and producing income share of total firm income in 2017
17. detect_largefarm: a binary indicator common to all firms that equals one if the ASF outbreak occurred in a large-scale (at least 500 pigs) pig farm
18. infected: the number of infected pigs reported from the ASF announcement
19. same_county: a binary indicator that equals one if the firm has at least one hog facility in a single county that reported an ASF outbreak
20. google: the daily number of searches for ‘African Swine Fever’ on Google
21. trade_cost: the differential trading costs from these food exporters to China by using a country-specific trade cost measure (Novy, 2013)

For the variables in the EXCEL file (ASF announcements.xlsx):

1. Relesae Date: defined the same as ‘asf_date’
2. Province_en: the province where the ASF outbreaks were confirmed
3. Prefecture (City): the prefecture where the ASF outbreaks were confirmed
4. County: the county where the ASF outbreaks were confirmed
5. Township: the township where the ASF outbreaks were confirmed
6. Place_type: where the ASF outbreaks were detected: hog farm, slaughterhouse, and transport.
7. Inventory: the hog inventory of the place where the ASF outbreaks were detected
8. Infected: the number of the infected hog at the place where the ASF outbreaks were detected
9. Death: the number of the dead hog at the place where the ASF outbreaks were detected

## 2 Experimental Design, Materials and Methods

‘Codes’ sub-folder contains the codes and a demo for event studies, the main research method in the supporting article, and codes for the pooled OLS regressions. Specifically, we use the ‘eventstudy2’ module to perform event study in this paper by Kaspereit [1]. ‘eventstudy2’ is not an official Stata command. This module should be installed from within Stata by typing "ssc install eventstudy2". It should be noted that we should first install other six user-written modules by typing “ssc install moremata”, “ssc install nearmrg”, “ssc install distinct”, “ssc install _gprod”, “ssc install rmse”, “ssc install parallel” before installing ‘eventstudy2’ module. For more information about ‘eventstudy2’ module, please type “help eventstudy2” in Stata.

The data and variables used in this DIB file mainly come from two sources: China Ministry of Agriculture and Rural Affairs (MARA), China (http://www.moa.gov.cn/gk/yjgl_1/yqfb/) (in Chinese) and Yahoo Finance, United States (https://finance.yahoo.com).

The Chinese MARA data source includes the nationally published events information for China's African Swine Fever outbreaks, and this information includes the specific date of a particular outbreak (asf_date), whether the outbreak was detected in a large pig farm (detect_largefarm), the place where the outbreak was detected (Place_type), the number of the hog inventory of the place, the number of infected pigs (infected), the number of dead pigs (infected).

All variables which appear in both ‘Chinese firms and market indices’ and ‘Global firms and market indices’ sub-folders are taken from Yahoo Finance, and none of the variables are created by the authors.

The variable “car” denoted the cumulative abnormal returns in the ‘Cumulative abnormal returns’ sub-folder. For firm *i* over a time interval $\tau =[\tau_{1},\tau_{2}]$ , we calculate CAR as

$$CAR_{i}\left(\tau_{1},\tau_{2}\right)=\sum_{t=\tau_{1}}^{\tau_{2}}AR_{it}$$

where $T_{2}+1\leq \tau_{1}\leq \tau_{2}\leq T_{3}$ and $AR_{it}$ is calculated from the event study. Please refer to Section 3 of the paper (Xiong et al., 2021).

The primary function of each file in this sub-folder is briefly introduced as follows.

[1] Codes_eventstudy.do: It's the main function that performs event studies.
[2] Event_id.dta: It's a STATA data file that contains the event's information.
[3] Marketfile.dta: It's a STATA data file that contains the stock market's information.
[4] Security_returns.dta: It's a STATA data file that contains the hog firms’ information.
[5] Codes_pooledols.do: It replicates the pooled OLS regression results in Tables 1 and 2, using STATA data files from the ‘Cumulative abnormal returns’ sub-folder.

**Table 1:** Regressions of Cumulative Abnormal Returns for Chinese Hog Firms.

Aug. 2018		Sept. 2018 — Dec. 2018	Jan. 2019 — Feb. 2019	Mar. 2019 — Sept. 2019	Aug. 2018 — Sept. 2019
Cumulative abnormal return*_t−_*_1_	1.0554***	0.9976***	0.9125***	0.9617***	0.9759***
	(0.0118)	(0.0041)	(0.0148)	(0.0064)	(0.0036)
Trading volume	0.8411*	0.5851***	0.9145***	0.4415***	0.5300***
	(0.4717)	(0.0832)	(0.0936)	(0.0419)	(0.0351)
Trading volume*t−*1	-0.6310	0.2139**	-0.3544***	-0.2285***	-0.2820***
	(0.4828)	(0.1027)	(0.0993)	(0.0430)	(0.0357)
Baidu searching index	0.0071***	0.0017*	0.0121*	0.0023	0.0024***
	(0.0019)	(0.0009)	(0.0062)	(0.0050)	(0.0008)
Baidu searching index*t−*1	-0.0042**	0.0028***	-0.0385***	0.0003	0.0003
	(0.0018)	(0.0009)	(0.0061)	(0.0050)	(0.0008)
Feeder pig price	-0.0034	-0.0013	0.0231***	0.0045***	0.0077***
	(0.0164)	(0.0023)	(0.0030)	(0.0011)	(0.0010)
Feeder pig price*t−*1	-0.0049	0.0017	-0.0302***	-0.0048***	-0.0089***
	(0.0151)	(0.0023)	(0.0031)	(0.0011)	(0.0010)
Hog breeding and producing income share	0.0043*	0.0007	-0.0092***	0.0007	0.0009
	(0.0025)	(0.0006)	(0.0032)	(0.0016)	(0.0006)
Was the outbreak detected in large-size pig farm?	-0.0007	-0.0007	0.0028	-0.0059	-0.0014**
	(0.0020)	(0.0005)	(0.0032)	(0.0037)	(0.0006)
The number of infected pigs	0.0022	-0.0006	0.0016	0.0017*	0.0003
	(0.0017)	(0.0004)	(0.0017)	(0.0009)	(0.0004)
Was the firm's pig farm in the same county as the outbreak?	-0.0001	0.0016**	-0.0033	0.0032*	0.0016**
	(0.0022)	(0.0006)	(0.0030)	(0.0018)	(0.0006)
Intercept	0.1619	-0.0524***	0.3664***	-0.0178*	-0.0172***
	(0.2935)	(0.0074)	(0.0433)	(0.0097)	(0.0056)

Observations	700	11900	1540	5180	19320
Adjusted R^2^	0.954	0.911	0.897	0.853	0.889
Number of firms	10	10	10	10	10
ASF events	5	85	11	37	138
Day dummies	Yes	Yes	Yes	Yes	Yes
Month dummies	No	No	No	No	Yes
Year dummies	No	No	No	No	Yes

Kao Test Statistics	-7.9119	-32.3250	-13.4389	-27.2926	-47.0227
Hadri LM Test Z Statistics	-0.5256	0.0279	-0.2504	-3.9384	-1.8488

Note: *** p *<* 0.01, ** p *<* 0.05, * p *<* 0.1. In all regressions, the dependent variables are cumulative abnormal returns (CAR). CAR values in all regressions are calculated using a 15-day event window. Day dummies indicate days since each ASF event, from day 1 to day 15. Kao test for panel cointegration uses the Augmented Dickey-Fuller t statistics with 1 lag, following the specifications in Equation (12); Hadri LM test for panel unit roots on residuals also includes 1 lag. Inference is based on clustered-robust standard errors (in parenthesis).

**Table 2:** Regressions of Cumulative Abnormal Returns for Foreign Hog Firms.

	Aug. 2018	Sept. 2018 — Dec. 2018	Jan. 2019 — Feb. 2019	Mar. 2019 — Sept. 2019	Aug. 2018 — Sept. 2019
Cumulative abnormal return*_t−_*_1_	1.0159***	0.9948***	0.9587***	1.0111***	0.9951***
	(0.0148)	(0.0056)	(0.0134)	(0.0062)	(0.0041)
Trading volume	0.0377	0.7796***	0.0409	0.2445*	0.5916***
	(0.4212)	(0.1675)	(0.0704)	(0.1445)	(0.1563)
Trading volume*t−*1	0.3629	-0.1995	0.5367***	0.3212**	-0.0112
	(0.4697)	(0.1338)	(0.1772)	(0.1401)	(0.1168)
Google searching index	2.86E-5	-2.53E-5***	-1.29E-5	-7.81E-6	-1.85E-5***
	(2.83E-5)	(9.15E-6)	(2.36E-5)	(1.14E-5)	(6.77E-6)
Google searching index*t−*1	-2.28E-5	-2.4E-5***	-9.16E-6	-2.1E-5*	–2.33E-5***
	(2.58E-5)	(8.83E-6)	(2.58E-5)	(1.15E-5)	(6.61E-6)
The number of infected pigs	4.04E-6	-0.0002	0.0003	0.0006	-0.0001
	(0.0009)	(0.0003)	(0.0005)	(0.0004)	(0.0002)
Trading cost from exporters to China	-0.0001**	-4.65E-5***	0.0001***	4.52E-5***	-1.6E-5
	(2.29E-5)	(1.18E-5)	(2.37E-5)	(1.39E-5)	(9.87E-6)
Intercept	0.0053	0.0039**	-0.0054*	-0.0061***	0.0039**
	(0.0040)	(0.0016)	(0.0032)	(0.0019)	(0.0016)

Observations	983	14907	2015	6332	24237
Adjusted R^2^	0.899	0.897	0.840	0.913	0.898
Number of firms	15	15	15	15	15
ASF events	5	85	11	37	138
Day dummies	Yes	Yes	Yes	Yes	Yes
Month dummies	No	No	No	No	Yes
Year dummies	No	No	No	No	Yes

Kao Test Statistics	-4.6772	-26.8848	-13.2969	-18.7947	-34.9042
Hadri LM Test Z Statistics	0.1924	0.0605	0.3058	0.2706	0.9391

Note: *** p *<* 0.01, ** p *<* 0.05, * p *<* 0.1. In all regressions, the dependent variables are cumulative abnormal returns (CAR). CAR values in all regressions are calculated using a 15-day event window. Day dummies indicate days since each ASF event, from day 1 to day 15. Kao test for panel cointegration uses the Augmented Dickey-Fuller t statistics with 1 lag, following the specifications in Equation (12); Hadri LM test for panel unit roots on residuals also includes 1 lag. Inference is based on clustered-robust standard errors (in parenthesis).

## Ethics Statements

N/A

## CRediT authorship contribution statement

**Tao Xiong:** Conceptualization, Methodology, Writing – review & editing. **Wendong Zhang:** Data curation, Writing – original draft, Visualization, Investigation. **Chen-Ti Chen:** Methodology, Formal analysis.

## Declaration of Competing Interest

The authors declare that they have no known competing financial interests or personal relationships that could have appeared to influence the work reported in this paper.

## Data Availability

Replication files for ``A Fortune from misfortune: Evidence from hog firms’ stock price responses to China's African Swine Fever outbreaks'' (Original data) (Mendeley Data).

## Footnotes

1 See Xiong et al. [3] for the full definition of the cumulative abnormal stock returns and the analyses conducted that use the variable, as well as the description of each research approaches in the supporting article.
